# Sex-specific winter distribution in a sexually dimorphic shorebird is explained by resource partitioning

**DOI:** 10.1002/ece3.1213

**Published:** 2014-10-02

**Authors:** Sjoerd Duijns, Jan A van Gils, Bernard Spaans, Job ten Horn, Maarten Brugge, Theunis Piersma

**Affiliations:** 1 Department of Marine Ecology, NIOZ, Royal Netherlands Institute for Sea ResearchP.O. Box 59, 1790 AB, Den Burg, Texel, The Netherlands; 2 Animal Ecology Group, Centre for Ecological and Evolutionary Studies, University of GroningenP.O. Box 11103, 9700 CC, Groningen, The Netherlands

**Keywords:** Bergmann's rule, habitat selection, intertidal ecology, *Limosa lapponica*, prey accessibility, sexual size dimorphism (SSD)

## Abstract

Sexual size dimorphism (SSD) implies correlated differences in energetic requirements and feeding opportunities, such that sexes will face different trade-offs in habitat selection. In seasonal migrants, this could result in a differential spatial distribution across the wintering range. To identify the ecological causes of sexual spatial segregation, we studied a sexually dimorphic shorebird, the bar-tailed godwit *Limosa lapponica*, in which females have a larger body and a longer bill than males. With respect to the trade-offs that these migratory shorebirds experience in their choice of wintering area, northern and colder wintering sites have the benefit of being closer to the Arctic breeding grounds. According to Bergmann's rule, the larger females should incur lower energetic costs per unit of body mass over males, helping them to winter in the cold. However, as the sexes have rather different bill lengths, differences in sex-specific wintering sites could also be due to the vertical distribution of their buried prey, that is, resource partitioning. Here, in a comparison between six main intertidal wintering areas across the entire winter range of the *lapponica* subspecies in northwest Europe, we show that the percentage of females between sites was not correlated with the cost of wintering, but was positively correlated with the biomass in the bottom layer and negatively with the biomass in the top layer. We conclude that resource partitioning, rather than relative expenditure advantages, best explains the differential spatial distribution of male and female bar-tailed godwits across northwest Europe.

## Introduction

Migratory animals need to acquire appropriate resources at multiple locations throughout their annual cycle (Alerstam and Lindström 1990; Newton 2008). Where populations occur over a large nonbreeding range, sites within that range may show different food regimes, weather conditions, levels of competition, and predation danger. Therefore, such migrants have to trade the costs (i.e., maintenance and migration costs) against the benefits (i.e., quality) of their alternative wintering sites (e.g., Drent and Piersma 1990; Castro et al. 1992; Alves et al. 2013a).

When ecological opportunities differ between classes of animals, such as sex, age, or subspecies, these classes may be expected to show different distributions (Cristol et al. 1999; Ruckstuhl 2007; Alves et al. 2012). Sexual size dimorphism (SSD) could result in males and females facing different trade-offs affecting migratory strategy and winter-site selection (Alves et al. 2013a), where the dominant sex may outcompete the other sex (e.g., Cristol et al. 1999; Blanckenhorn 2005). Indeed, segregation between the sexes during the nonbreeding season has been documented for some migratory birds at different spatial scales (e.g., Ketterson and Nolan 1976; Myers 1981; Mathot et al. 2007; Nebel et al. 2013).

When individuals differ in body size, they will not only differ in energetic requirements but also in the use of a given resource. Such resource partitioning can lead to spatial segregation (Schoener 1974). In many bird species, bill size is a strong predictor of foraging niche (Selander 1966) and differences in bill structure and size will be associated with differences in feeding technique and diet (Rubega 1996; Durell 2000). Thus, sexual differences in bill morphology might lead to sex differences in diets related to prey size or prey burying depth (Mathot et al. 2007; Alves et al. 2013b; Duijns and Piersma 2014).

In this study, we examine wintering site selection for a long-distance migrating sexually dimorphic shorebird, the bar-tailed godwit *Limosa lapponica lapponica*. This subspecies breeds in northern Scandinavia and winters almost exclusively in Europe (Duijns et al. 2012). Sexual dimorphism is most pronounced in body size and bill length, with females being 20% larger and having 25% longer bills than males (e.g., Piersma and Jukema 1990; Duijns et al. 2012). Within the wintering range of this population, spatial segregation between the sexes has been observed. The smaller males occur in climatically mild areas such as the United Kingdom (Atkinson 1996; Summers et al. 2013), whereas most females are found in the northern and colder parts of the European Wadden Sea (Smith 1975; Prokosch 1988; Scheiffarth 2001a). It has been hypothesized that the high living costs at sites closer to the breeding areas may be energetically more advantageous for the larger sex (Smith 1975; Scheiffarth 2001a). One of the best-known ecological generalizations with respect to large-scale distributions of species is Bergmann's rule (1847). This rule states that within a genus of endothermic vertebrates, the larger variants will be found in cooler environments as they have lower surface to volume ratios and will proportionally radiate less heat per unit body mass.

**Figure 1:**
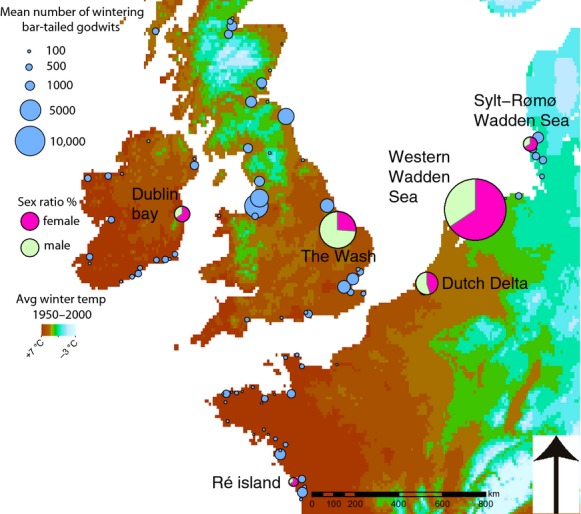
Map of North-western Europe, encompassing all wintering sites of bar-tailed godwits. Location of the study sites, with the mean January numbers of bar-tailed godwits (1995–2005) counted at high-tide roosts, based on the Wetlands International midwinter count database. Mean winter temperature data (1950–2000), of high spatial resolution, were derived from satellite images through interpolation of climate data (Hijmans et al. 2005). There is a clear gradient in temperature from Sylt-Rømø Wadden Sea to the Dutch Western Wadden Sea, to the UK and Ireland and southern wintering areas in France.

Alternatively, for shorebirds that feed in soft substrates, shorter-billed birds may rely more heavily on shallowly buried prey from the sediment surface compared to longer-billed birds, which are able to probe more deeply into the sediment to extract more deeply buried prey (e.g., van de Kam et al. 2004; Mathot et al. 2007). Benthic organisms are distributed throughout intertidal sediment with the larger and more profitable prey (e.g., Alves et al. 2013b; Duijns and Piersma 2014) found deeper and the smaller prey occurring closer to the surface (Reading and McGrorty 1978; Zwarts and Wanink 1991). Indeed, bar-tailed godwit diet composition differs between the sexes, where the shorter-billed males frequently feed on the smaller and shallowly buried prey, and the longer-billed females predominantly feed on the larger and more deeply buried prey (Scheiffarth 2001b; Duijns and Piersma 2014). This would suggest that the shorter-billed males should spend the non-breeding season at sites with a high density of food items available at or near the surface, whereas the longer-billed females should winter in areas with a high density of deeper buried prey. To address the mechanisms underlying this sex-specific spatial pattern, we have quantified the occurrence of these shorebirds and benthic prey availability at six important non-breeding sites across the wintering range in Western Europe (Fig.1).

## Methods

### Study sites

Field work was carried out at six nonbreeding sites. The initial choice for the sites was based on the top ten highest mean January counts from 1995–2005, as obtained from the Wetlands International midwinter count database. In only six areas, numbers seemed high enough and logistics were favorable. The sites were located throughout Western Europe, spanning 1200 km and 14° of longitude and 11° of latitude. Although we visited the sites in as brief a period as possible, measurements could not be made simultaneously. However, individual shorebirds that have selected a wintering site are known to be site-faithful (e.g., Burton 2000; Leyrer et al. 2006), and benthic prey availability was shown to be relatively constant during the winter months (Zwarts and Wanink 1993). The German Sylt-Rømø Wadden Sea area (55°01′N, 8°26′E) was visited in mid-October 2010, the Dutch Delta area (51°40′N, 04°07′E) in late October 2010, the Wash in the UK (52°56′N, 00°19′E) in early November 2010, Dublin bay in Ireland (53°19′N, 06°11′W) in late November 2010, the Western Wadden Sea island Griend (53°14′N, 05°15′E) in early February 2011, and Ré island (46°15′N, 01°29′W) in France in late December 2013. See Table1 for more details on the study sites.

**Table 1:** Main characteristics of the study sites, including distance to the breeding grounds, benthic biomass (distinguished in ash-free dry mass (AFDM) of top and bottom layer and percentage of AFDM in the bottom layer), and mean winter temperature

Location	Distance (km) to breeding grounds	Biomass top layer (0–4 cm) g AFDM·m^−2^ ± SD	Biomass bottom layer (5–30 cm) g AFDM·m^−2^ ± SD	Percentage of AFDM ± SD in the bottom layer	Mean winter temperature (°C)
Sylt-Rømø Wadden Sea	1940	1.73 ± 0.94	14.85 ± 13.40	80.09 ± 24.07	0
Western Wadden Sea	2196	1.42 ± 1.35	8.06 ± 5.32	89.02 ± 9.65	2
The Wash	2357	1.58 ± 1.31	2.22 ± 1.94	64.99 ± 16.98	4
Dutch Delta	2388	1.11 ± 0.67	7.82 ± 6.58	75.28 ± 25.61	3
Dublin bay	2502	0.47 ± 0.65	7.35 ± 2.96	93.25 ± 10.13	5
Ré island	3093	0.39 ± 0.26	1.75 ± 2.26	80.79 ± 22.33	7

### Sex-ratio counts

At each study site, multiple sex-ratio counts were made. On average, a count covered 117 ± 108.4 SD individuals (*N* = 61) and sex ratios are expressed as % females. As bar-tailed godwits show such a strong sexual dimorphism, the sex of each bird could easily be distinguished in the field on the basis of overall body size dimensions (see Zwarts et al. 1990; Scheiffarth 2001a), and all birds were observed in full winter (basic) plumage. Each flock was scanned by initiating a count with a randomly chosen individual and then by moving away either always left or right from the first bird. This ensured that the same individual was not counted twice. We also noted the abdominal profile score per sex (ranging from 1 – lean – to 5 – abdomen bulging), to estimate body condition (Wiersma and Piersma 1995; Duijns et al. 2009), as individuals wintering at more northerly (and thus colder) sites are expected to increase energy stores (sensu Lindström and Piersma 1993) to survive days that food may not be accessible at all (e.g., the freezing over of mudflats in the Wadden Sea, see Zwarts et al. 1996). New counts were made when flocks arrived or departed. We validated our visual estimates of sex by assigning marked individuals of known sex in the field, based on morphological measurements (Prater et al. 1977) at different distances (20–150 m) and locations, prior to this study. That we correctly could assign 354 marked individuals of 364 sightings (97.3%), suggests that our observational sex assignments were robust.

### Benthic food availability

At locations where we observed (>30 min) foraging flocks of bar-tailed godwits, 10 randomly located benthic samples were taken. Each sample consisted of a core of 0.0177 m^2^ to a depth of 30 cm, which was sieved through a 1 mm mesh. Note that some prey items such as the lugworm *Arenicola marina*, a preferred prey for female bar-tailed godwits, can live up to depths of 30 cm. In order to split prey availability into shallow and deep prey, we sieved the top 4 cm separately from the rest of the sample. The reason for separating prey availability in top and bottom in this manner was threefold. (1) From previous work on this species (Duijns and Piersma 2014), it was found that males were more successful in finding prey items after pecking, whereas females are most successful in finding prey items after probing (Table2). Pecks include all behaviors that involve contact of the bill to the sediment surface (i.e., approx. 3–4 cm), and probes included all behaviors involving insertion at least 1/3 of the bill into the sediment. (2) Separating the top 4 cm from the bottom part of the core has been the standard approach in the last two decades within our research group (Piersma et al. 2001; van Gils et al. 2006; Kraan et al. 2009), enabling us to compare the benthic food abundances between different areas. (3) As this species' diet comprises mostly polychaetes (Duijns et al. 2013), which are mobile and can move through the sediment (Duijns and Piersma 2014), separating the benthic sample in more layers would result in many prey to break, making it impossible to distinguish in which layer they would predominantly occur.

**Table 2:** Percentage of successful pecks and probes for male and female bar-tailed godwits observed in the Dutch Wadden Sea (Duijns and Piersma 2014)

	% Successful pecks	*N*	% Successful probes	*N*
Males	67	425	33	120
Females	13	42	87	124

All prey items were counted per species and stored in a 4% formaldehyde saline solution for later analyses. To determine the ash-free dry mass (AFDM; g), prey items were dried to constant mass in a ventilated oven at 55–60°C, after which dry mass was determined. The dried flesh of all species was incinerated at 560°C for 5 h. The remaining ash mass was then subtracted from the dry mass to determine the AFDM (Table1).

### Maintenance energy requirements

The maintenance energy requirements (*M*_maint_) were calculated as basal metabolic rate (BMR; W) plus extra costs for thermoregulation (i.e., standardized heat loss, *H*_sm_; W) at environmental temperatures:


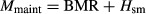
(1)

where BMR per sex was calculated using the equation for shorebirds wintering in temperate Europe (Kersten and Piersma 1987):


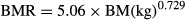
(2)

in which body mass (BM, kg) was taken as the mean of winter catches at 0.270 and 0.323 kg for males and females, respectively (NIOZ unpublished data). The standardized heat loss (*H*_sm_) was calculated using Wiersma and Piersma's (1994) equation:


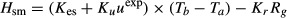
(3)

where *K*_es_ represents the thermal conductance of a live bird (W°C^−1^), which was sex specific (0.0914 for males and 0.1111 for females, see Scheiffarth et al. 2002); The coefficients *K*_u_ and *K*_r_, as well as the exponent for wind speed (*exp*), were based on the iterative regression procedure from Scheiffarth et al. 2002; *u* denotes the average winter wind speed (m·s^−1^), as obtained from the European Climate Assessment & Dataset project (http://www.eca.knmi.nl); *T*_b_ represents body temperature (°C), which was assumed to be equal for both sexes (i.e., 41°C); *T*_a_ represents the mean winter temperature (°C; October to March), as derived from weather stations (Hijmans et al. 2005) based long-term averages (1950–2000) and *R*_g_ represents the mean winter global radiation (W·m^−2^) as obtained from SoDa (http://www.soda-is.com).

### Migration costs

Flight distances (km) between wintering sites and a fixed site in the breeding grounds in Norway (70°16′N, 24°05′E; Aarvak and Oien 2009) were measured using the distance tool in Google Earth ver. 7.1.2 (http://www.google.com/earth/) and multiplied by 2. This web-based software measures distances in great circle lines (or orthodrome lines), which are the shortest routes between two points on the globe (Alves et al. 2012). The migration costs (C_flight_; kJ) per sex were calculated using the following equation:


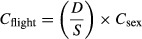
(4)

where the distance (D; km) is divided between the average flight speed (*S*) of 75 km·h^−1^ and a sex-specific empirical flight cost (*C*_sex_) of 67 and 55 kJ·h^−1^ for females and males, respectively (Piersma and Jukema 1990).

### Statistical analyses

The frequency of occurrence of male and female bar-tailed godwits per area was analyzed with linear mixed models (LMMs), where the response variable proportion of sex per observation session was logit-transformed (Warton and Hui 2011), the explanatory variable was study site, and observation session was the random effect. Differences in top and bottom layer biomass were analyzed with a general linear model (GLM), and a Tukey's test was used to detect differences between sites. A Pearson correlation coefficient (*r*) was used to determine the relationship between the available biomass in the top and bottom layers and to determine the correlation between costs and benefits (i.e., food availability separated in top and bottom layer) and the % females per area. All analyses were performed using R, version 3.1.0 (R Development Core Team 2014), and the package lme4 (Bates et al. 2013) was used to fit linear mixed models.

## Results

### Large-scale sexual segregation

**Figure 2:**
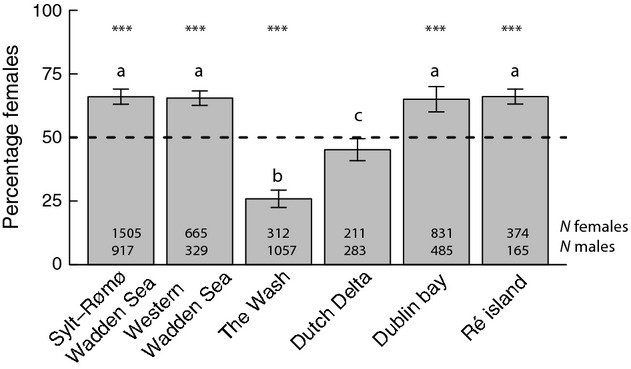
Geographical variation of the mean percentage (95% CI) of female bar-tailed godwits. The dashed line indicates a balanced sex ratio. Letters refer to the differences between the different sites and asterisks above the figure refer to significantly biased sex ratios within areas (all *P* < 0.001). Sample sizes of total number of birds counted per sex are given inside the bars.

The sexes were differentially distributed over the six different sites across North-western Europe (LMM, *χ*^*2*^ = 57.81, df = 5, *P <* 0.001; Fig.2). The Wash and the Dutch Delta area were different from the other four sites (Tukey's test, *P* < 0.05). Relatively more males were found in The Wash, while in Dublin bay, Sylt-Rømø Wadden Sea, Ré island and the Western Wadden Sea, a higher proportion of females was present (Fig.2).

### Resource abundance

**Figure 3:**
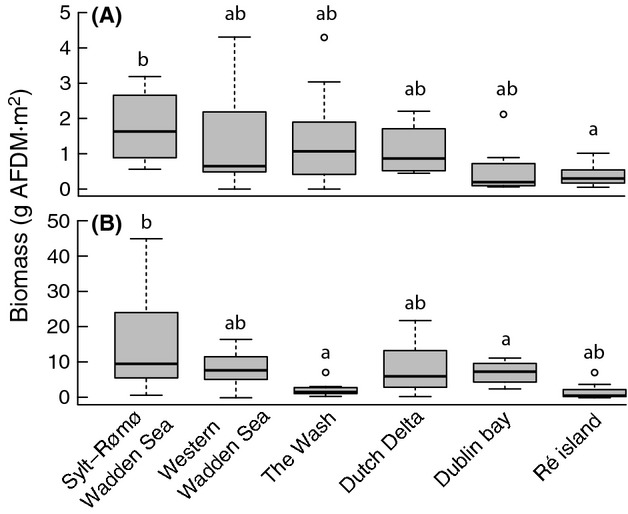
Biomass of (A) top 0–4 cm and (B) bottom 5–30 cm layer, as expressed in ash-free dry mass (g AFDM·m^−2^). The letters denote significance levels (Tukey's test), where the top layer of the Sylt-Rømø Wadden Sea area is different from Ré island and the bottom layer from the Sylt-Rømø Wadden Sea area is different from Dublin bay and the Wash.

Study sites differed in prey biomass (i.e., g AFDM·m^−2^) in the top (ANOVA, *F*_5,52_ = 3.725, *P =* 0.006; Fig.3A) and in the bottom layer (ANOVA, *F*_5,54_ = 4.998, *P <* 0.001; Fig.3B). However, due to high variation within sites, the difference was only due to the relatively high food abundance in the Sylt-Rømø Wadden Sea area, both for top as well as for bottom layer (Tukey's test, *P* < 0.05).

### Maintenance and migration costs

**Figure 4:**
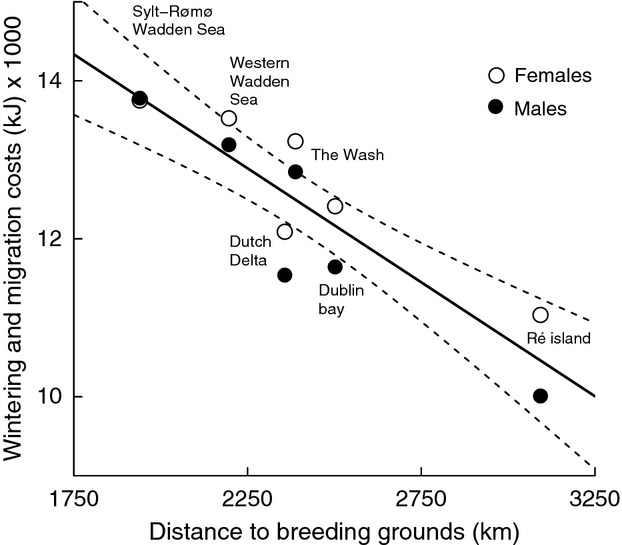
Relation between the costs of wintering (including the migration costs) in relation to the distance to their breeding grounds (the dashed lines represent the 95% CI level) for male and female bar-tailed godwits. There is a negative relation between the cost and the distance to the breeding grounds for both sexes (*P* < 0.001). Note that the difference in costs between females and males increases with increasing distance. This is consistent with Bergmann's rule as the thermoregulatory benefits of wintering further south increase fastest for the smallest sex (losing more heat per unit body mass).

**Figure 5:**
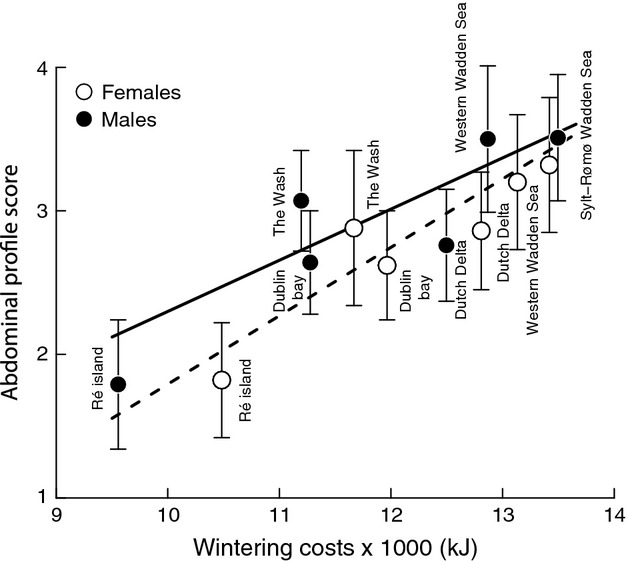
Mean abdominal profile score increases with increasing wintering costs, with significant differences between the sexes (*P* = 0.006; dashed line represents the males and the solid line the females) and a significant interaction term (*P* = 0.016).

The maintenance costs decreased in a linear fashion with increasing distance from the breeding grounds, (*F*_1,10_ = 39.75, *P* < 0.001, *R*^2^ = 0.79), with no difference between the sexes. Additionally, the cost of migration, at about 3% of the maintenance costs, turned out to be small and not affecting the overall picture. Hence, costs of wintering including the cost of migration also decreased linearly with increasing distance from the breeding grounds (*F*_1,10_ = 48.03, *P* < 0.001, *R*^2^ = 0.83; Fig.4). The abdominal profile scores suggest that male and female bar-tailed godwits did indeed adjust body mass to the costs of wintering (Fig.5), with the males opting for a higher relative level of energy stores than females (*F*_3,585_ = 105, *P* = 0.006, *R*^2^ = 0.35), with a significant interaction between sex and the cost of wintering (*P* = 0.016).

### Resource partitioning versus maintenance and migration costs

**Figure 6:**
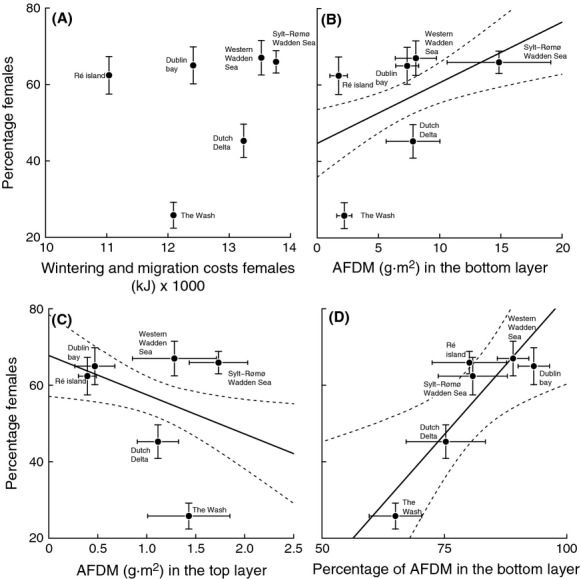
(A) Cost of wintering at different wintering site does not correlate with the percentage of females. (B) The (absolute) food abundance in the bottom layer (5–30 cm) is positively correlated with the percentage of females (the dashed lines represent the 95% CI level), (C) and the (absolute) food abundance in the top layer (0–4 cm) is negatively correlated with the percentage of females. (D) The percentage of AFDM in the bottom layer is positively correlated with the percentage of females.

The percentage of females wintering at a given site was not correlated with wintering costs (*r* = 0.22, df = 4, *P* = 0.67; Fig.6A). Despite the fact that the biomass (g AFDM·m^−2^) in the top and bottom layer were positively correlated (*r* = 0.50, *N* = 60, *P* < 0.001), the percentage of females was only positively correlated with the biomass in the bottom layer (*r* = 0.38, df = 59, *P* = 0.002; Fig.6B) and negatively with the biomass in the top layer (*r* = −0.29, df = 59, *P* = 0.002; Fig.6C). There was a strong positive correlation between the percentage females and the percentage of AFDM in the bottom layer (*r* = 0.88, df = 4, *P* = 0.02; Fig.6D). These patterns are consistent with the resource partitioning hypothesis.

## Discussion

In this study, we show evidence for resource partitioning between the sexes of a migratory shorebird with respect to their differential winter distribution at a large scale. At the level of sites separated by at least 200 km, we observed an unequal distribution of the sexes and could link this to the availability and vertical distribution of their benthic prey. Any relatively lower costs for the larger sex wintering closer to the breeding areas (according to Bergmann's rule) would surely be overridden by the fact that at the northerly sites food availability for the larger sex was much higher than for the smaller sex. Therefore, the present study suggests that at this scale the birds go where the food is most available to them. This was previously found in a species bar-tailed godwits share the general habitat with, but eating molluscs rather than polychaetes, the red knot *Calidris canutus* (e.g., van Gils et al. 2004; Quaintenne et al. 2011; Piersma 2012).

The uneven distribution between the sexes found in this study corresponded with data collected in a similar fashion at the Sylt-Rømø Wadden Sea area (Scheiffarth 2001a), Ré Island (P. Bocher, pers. obs), and in previous years at the Dutch Wadden Sea (S. Duijns pers. obs). Also at the Wash, where birds were caught by cannon nets, were the sex ratios consistent with our study (Atkinson 1996). The results of this study therefore reveal a temporally consistent pattern.

Our results provide an interesting contrast with data on sex-related differences in coastal habitat use in a congener, the Icelandic black-tailed godwits *Limosa limosa islandica* (Alves et al. 2013b). Here, over the entire winter range during the nonbreeding season, no evidence of large-scale sex differential distribution was found, when compared to seasonal population estimates of sex ratios. The sexes differed in their selection of prey types and sizes, leading to small-scale sexual segregation within, rather than between estuaries. In bar-tailed godwits, such small-scale segregation between male and females also exists and was documented for coastal Guinea-Bissau (Zwarts 1988), in the UK (Smith and Evans 1973; Summers et al. 2013), the western Wadden Sea (Both et al. 2003), and in France (P. Bocher, pers. obs). That females seemed more abundant than males at the sampled sites is unlikely due to a biased overall sex ratio, because unbalanced wild bird populations tend to be male-skewed rather than female-skewed (Donald 2007).

These results, however, do not mean there are no expenditure-related costs of wintering close to the breeding grounds. That male and female bar-tailed godwits adjust their body mass with the males opting for a relatively higher level of energy stores than females suggests that they may need a larger safety margin because they would face a greater risk of being without food than females (e.g., MacLeod et al. 2006, 2007). Due to their larger surface to volume ratios, males will also have more variable energy expenditures between days. If we interpret the levels of stores as indication of higher costs carried due to risk aversion, or buffering against lower quality habitat (Macleod et al. 2008), males might thus be in less favorable habitats. Their greater nutrient stores would enable them to survive periods of unpredictable food resources (Rogers 1987).

Wintering closer to the breeding grounds could facilitate the timing of migration, as residing closer to breeding area, local weather systems may promote an advantageous migratory flight strategy (Piersma et al. 1994). However, escape performance in birds generally is reduced by extra body mass, as it leads to a decrease in take-off speed and maneuvrability (e.g., Dietz et al. 2007). As both sexes, based on their abdominal scores (Fig.5), have a larger antistarvation safety margin at colder sites, it could make both sexes more susceptible for predation there. Note that we never witnessed any attacks by aerial predators.

In conclusion, the resource partitioning hypothesis best explained the distribution between the sexes, where the larger females may have a subtle benefit of wintering close to the breeding area, as their relatively smaller stores suggest a lower risk of starvation relative to males.
